# The Role of Adenosine Signaling in Headache: A Review

**DOI:** 10.3390/brainsci7030030

**Published:** 2017-03-13

**Authors:** Nathan T. Fried, Melanie B. Elliott, Michael L. Oshinsky

**Affiliations:** 1 Department of Neurology, Thomas Jefferson University, Philadelphia, PA 19107, USA; nathanth@mail.med.upenn.edu; 2 Department of Neuroscience, University of Pennsylvania, Philadelphia, PA 19104, USA; 3 Department of Neurosurgery, Thomas Jefferson University, Philadelphia, PA 19107, USA; melanie.elliott@jefferson.edu; 4 National Institutes of Health, National Institute of Neurological Disorders and Stroke, Bethesda, MD 20892, USA

**Keywords:** migraine, headache, adenosine, adenosine receptors, mitochondria, astrocytes, trigeminal

## Abstract

Migraine is the third most prevalent disease on the planet, yet our understanding of its mechanisms and pathophysiology is surprisingly incomplete. Recent studies have built upon decades of evidence that adenosine, a purine nucleoside that can act as a neuromodulator, is involved in pain transmission and sensitization. Clinical evidence and rodent studies have suggested that adenosine signaling also plays a critical role in migraine headache. This is further supported by the widespread use of caffeine, an adenosine receptor antagonist, in several headache treatments. In this review, we highlight evidence that supports the involvement of adenosine signaling in different forms of headache, headache triggers, and basic headache physiology. This evidence supports adenosine A_2A_ receptors as a critical adenosine receptor subtype involved in headache pain. Adenosine A_2A_ receptor signaling may contribute to headache via the modulation of intracellular Cyclic adenosine monophosphate (cAMP) production or 5' AMP-activated protein kinase (AMPK) activity in neurons and glia to affect glutamatergic synaptic transmission within the brainstem. This evidence supports the further study of adenosine signaling in headache and potentially illuminates it as a novel therapeutic target for migraine.

## 1. Introduction

Adenosine is a purine nucleoside that plays a critical role in numerous cellular and molecular functions throughout the brain such as metabolism, cell signaling, purinergic neuronal signaling, and inflammation [1]. Not surprisingly, adenosine is appreciated for its complex role in many nervous system disorders such as alcoholism, epilepsy, traumatic brain injury, ischemia, anxiety, Alzheimer’s disease, Parkinson’s, and brain cancer [2,3,4,5,6,7,8]. Although progress has been made in understanding adenosine’s role in certain pain states, less is known about how it is involved in migraine [9].

Current treatment options for headache and migraine include analgesics, triptans, ergots, behavioral intervention, and nerve stimulation, while new treatments, such as monoclonal antibodies against Calcitonin gene-related peptide (CGRP) or the CGRP receptor and CGRP inhibitors, are under development. Some patients who are non-responders to these treatments choose to undergo surgical implantation of invasive nerve stimulators to relieve pain [10]. A component of some of these treatment options is caffeine, a non-specific adenosine receptor antagonist [11]. Combining caffeine with other treatments can improve pain relief by as much as 40% in patients [12,13,14,15,16]. Caffeine was originally included in many migraine treatments due to the previous assumption that migraine was a vascular disorder. Now that migraine is thought to be a neurological and not a vascular disorder, caffeine’s common inclusion in migraine treatments suggests an alternative, non-vascular mechanism of action behind its efficacy in migraine patients. In this review, we present mounting evidence that demonstrates adenosine’s role in migraine and headache, revealing it as a potential therapeutic target for migraine and headache treatment.

## 2. Modeling Headache and Migraine

Headache is described as pain located above the orbitomeatal line that transects the head, approximately from the top of the eyes to the back of the skull. Migraine is a disabling neurological disorder most often characterized by the accompanying headache, but it is much more than just an episodic headache as it includes many other symptoms such as phonophobia, photophobia, nausea, and visual auras during the prodromal phase in a subset of patients. Thirty-six million individuals in the United States suffer from migraine, with a cost-estimate of $50 billion each year [17]. The World Health Organization has classified it as the third most common disease worldwide [18]. Fourteen-million migraine patients are diagnosed as chronic migraineurs, described as an individual who experiences a migraine at least fifteen days out of every month for at least three months [17].

The headache phase of migraine is thought to be caused by activation of dural C and Aδ nociceptors of the trigeminal ganglion [19]. However, no direct evidence for this exists due to the difficulty of studying the contribution of meningeal afferents to headache in humans. However, functional magnetic resonance imaging data demonstrating increased activity in the trigeminal ganglion during migraine as well as other human studies suggest that headache pain is transmitted via these nociceptors [20,21]. Trigeminal afferent fibers project centrally to second order neurons of the trigeminal nucleus caudalis (TNC) in the brainstem. It is thought that peripheral and central changes occur after repeated episodic nociceptor activation that sensitizes this trigeminal system, exacerbating the perception of migraine pain in secondary or referred areas [22]. Chronic migraine most likely involves the development of chronic peripheral and central sensitization from repeated attacks [23,24].

We have previously developed two behavioral animal models of chronic trigeminal allodynia that feature numerous migraine-like characteristics to allow for the investigation of migraine pathophysiology [25,26,27,28,29,30,31]. The first model, designated as the inflammatory stimulation model (IS rats), was developed by simulating the putative dural nociceptor activation thought to occur during a headache attack by infusing an inflammatory soup (1-mM histamine, serotonin, bradykinin, and 0.1-mM prostaglandin E_2_ in phosphate-buffered saline (PBS), pH 7.4) onto the dura through an affixed cannula 3 ×/week for a total of 12 infusions [26]. Each infusion causes the development of trigeminal sensitivity in the periorbital region, as measured with von Frey hairs, which recovers within 2–3 h. Following approximately the fifth infusion, the animals’ baseline thresholds begin to also decrease (i.e., the animal is still experiencing trigeminal sensitivity before the following infusion is performed). This repeated inflammatory dural stimulation then induces a steady-state of chronic trigeminal sensitivity that outlasts the final infusion for months.

The second model, called the spontaneous trigeminal allodynia (STA) in rats, is currently the only model of primary trigeminal allodynia in animals [32]. Naturally born with this trait, no surgical procedure or pharmacological treatment is needed to produce an allodynic state that is confined to the trigeminal system. These animals are not genetically modified, but were instead discovered and bred to create a colony of STA rats. The gene or genes responsible for this trait are currently unknown. Similar to human migraineurs who experience a combination of headache-free and headache days with fluctuating intensity, these animals feature episodically fluctuating trigeminal sensitivity. Both models of headache share symptomology similar to migraine in that they experience phonophobia, sensitivity to migraine triggers, similar efficacious responses to migraine treatments, and trigeminal sensitivity (although expressed differently—IS rats with stable chronic trigeminal sensitivity and STA rats with episodically fluctuating chronic trigeminal sensitivity) [25,27,28,29]. These animal models provide an experimental means to determine elements that are key to migraine pathophysiology and to screen potential therapeutic treatments.

## 3. Adenosine Receptor Signaling in the Nervous System

The physiological role of adenosine was first appreciated in 1929 when it was described for its ability to modulate the beat and conductance of cardiac muscle fibers [33]. Adenosine is now well-accepted as a neuromodulator with extracellular levels of it derived from several cellular sources within the central nervous system, including multiple glia and neuronal cell types [34,35,36,37]. Some evidence even suggests it may be directly packaged and released as a neuro/glio-transmitter in a Soluble NSF Attachment Protein Receptor (SNARE)- and stimulus-dependent manner [38,39,40]. Similar to a neuro/glio-transmitter, there are distinct mechanisms for the removal of adenosine from the extracellular space to deactivate its effects, such as reuptake via equilibrative nucleoside transporters (ENTs) or metabolism to inosine by adenosine deaminase (ADO) [41,42]. Three primary mechanisms are responsible for the production/release of extracellular adenosine: equilibrative transport of adenosine out of the cell through equilibrative nucleoside transporters (ENTs) when intracellular levels are high [43], metabolism of released adenosine triphosphate (ATP) to adenosine by subsequent hydrolysis steps with extracellular 5′ ectonucleotidases (i.e., ATP to adenosine diosphate (ADP) to adenosine monosphate (AMP) to adenosine) [44], or activity-dependent mechanisms during gliotransmission [38,39,40].

There are four known adenosine receptor subtypes. These purinergic G protein-coupled receptors each modulate adenylate cyclase activity in alternate ways [1]. Adenosine A_2A_ and A_2B_ receptors are coupled to the G_s_ alpha subunit, stimulating adenylate cyclase activity, while adenosine A_1_ and A_3_ receptors are coupled to the G_i_ alpha subunit, inhibiting adenylate cyclase activity [45]. Control of adenylate cyclase activity with these receptors allows adenosine to fine-tune intracellular cAMP levels to control a large host of cellular mechanisms including resting potentials, metabolism, calcium channel phosphorylation, and gene expression [46]. Each adenosine receptor subtype has been reported to be expressed throughout the central and peripheral nervous system. Adenosine A_1_ receptors are the most abundant adenosine receptors in the brain and are found in the neocortex, cerebellum, hippocampus, and the dorsal horn [45,47]. Adenosine A_2A_ receptors are more widely distributed throughout the entire brain and the peripheral nervous system; they are found on pre- and postsynaptic nerve terminals, astrocytes, and the blood-brain barrier [45,48,49]. Adenosine A_2B_ receptors are less studied but are present on many cell types including astrocytes [50,51,52]. Adenosine A_3_ receptor expression has been reported throughout the brain with a particular abundance in the hippocampus [53,54,55,56]. With this wide expression of multiple adenosine receptor subtypes throughout the brain, it is thought that adenosine subtype-specific physiological processes are achieved by their different affinities for adenosine: A_1_, 3–30 nM; A_2A_, 1–20 nM; A_2B_, 5–20 µM; A_3_, >1 µM [57]. Thus, localized modulation of adenosine concentrations would dictate subtype specificity.

Basal extracellular adenosine levels have been estimated in rats with microdialysis and range between 30–970 nM depending on the area of the brain [57]: striatum (80 nM) [58], cortex (120 nM) [59], basal forebrain (30 nM) [60], hippocampus (850 nM) [61], and thalamus (970 nM) [62]. Microdialysis, however, is heavily dependent on the time point at which the samples are collected. Insertion of the microdialysis probe causes damage that leads to a large increase in extracellular neurotransmitter levels. An acclimation period of at least two hours is needed to establish a baseline before measuring neurotransmitter concentrations [29,63]. This is seen in the above estimates in the hippocampus and thalamus which are taken prior to the 2 h-post implantation time point. In fact, other forms of adenosine measurement such as the collection of whole brain samples or cerebral spinal fluid and in vitro pharmaco-dynamic measurement of adenosine suggests lower basal adenosine levels of 50–200 nM [57]. Although these estimates of basal adenosine levels are high enough to activate A_1_ and A_2A_ receptors, it is important to note that these techniques do not allow for the measurement of adenosine concentration in the synaptic cleft or at the site of the receptor which may be highly regulated by ENTs and adenosine deaminase.

## 4. The Role of Adenosine in Pain

The investigation of adenosine’s potential as a therapeutic has revealed its complex role in pain. Adenosine signaling can be anti- or pro-nociceptive depending on the location (central or peripheral), form of pain (acute or chronic), and the adenosine receptor subtype targeted [64,65].

Adenosine A_1_ receptor agonists are anti-nociceptive [66]. Adenosine A_1_ receptor activation in peripheral nerve terminals with N6-cyclopentyladenosine (CPA) prevented prostaglandin E_2_-induced mechanical allodynia in the hindpaw of rats [67]. Adenosine A_1_ receptor agonists, R-phenylisopropyl-adenosine and *N*-ethylcarboxamide-adenosine, decreased formalin-induced hyperalgesia in rats (as measured by total licking time during a five minute period) [68]. Mice genetically engineered to lack adenosine A_1_ receptors had enhanced thermal hyperalgesia and lost the analgesic effect of intrathecal adenosine injection [69]. Administration of CPA, the adenosine A_1_ receptor agonist, decreased thermal hyperalgesia in normal rats and decreased both mechanical and thermal hyperalgesia in nerve injured rats. These analgesic effects were reversed with administration of the adenosine A_1_ receptor antagonist, 8-cyclopentyl-1,3-dipropylxanthine (DPCPX) [70]. Adenosine A_1_ receptors have been found to play a vital role in the adenosine-induced antinociceptive effects of acupuncture in mice where adenosine A_1_ knock-out mice did not experience the anti-nociceptive effects of acupuncture in an inflammatory or neuropathic pain model [71]. Intrathecal injection of an adenosine A_1_ agonist in humans decreased postoperative pain [72]. Interestingly, intrathecal injection of adenosine (i.e., non-subtype specific action) prevents post-operative pain but causes headache, suggesting that other adenosine receptor subtypes may play an opposing role in headache when compared to other forms of pain [73,74]. 

Adenosine A_2A_ receptor agonists are pro-nociceptive [66]. Adenosine injection into the hindpaw of rats induced a dose-dependent increase in mechanical hyperalgesia in a cAMP-mediated manner. This was prevented by the administration of the adenosine A_2A_ receptor antagonist, PD 081360-0002. In the same study, a hyperalgesic response similar to adenosine injection was produced with the injection of adenosine A_2A_ receptor agonists, 5′-(*N*-ethyl)-carboxamido-adenosine or 2-phenylaminoadenosine [67]. The adenosine A_2A_ receptor agonist, 2-p-(2-carboxyethyl) phenethylamino-5′-*N*-ethylcarboxamido adenosine hydrochloride (CGS-21680), enhanced formalin-induced pain in rats while the adenosine A_2A_ antagonist, 3,7-dimethyl-1-propargylxanthine (DMPX), decreased formalin-induced pain [75]. Another adenosine A_2A_ agonist, 2-(2-aminoethylamino)-carbonylethylphenylethylamino-adenosine (APEC), enhanced formalin induced pain in mice [68]. Microdialysis in the rat hindpaw demonstrated that subcutaneous formalin induces a dose-dependent increase in adenosine production, further suggesting adenosine involvement in formalin-induced pain [76]. An adenosine A_2A_ knock-out mouse had a decreased thermal pain response, suggesting that the presence of adenosine A_2A_ receptors promotes thermal pain [77]. These knock-out mice also feature increased mechanical hyperalgesia in response to inflammatory-induced pain [78].

Less is known about the specificity of adenosine A_2B_ and A_3_ receptors in pain. Adenosine A_2B_ receptor activation, increasing intracellular cAMP levels similar to adenosine A_2A_ receptor activation, appear to be pro-nociceptive. Administration of multiple adenosine A_2B_ antagonists prevented thermal-induced pain in mice [79]. Mechanical and thermal pain was enhanced in an adenosine deaminase (ADO) knock-out mouse, leading to system-wide adenosine elevation. This pain was prevented pharmacologically with the adenosine A_2B_ antagonist, PSB-1115 (1-propyl-8-p-sulfophenulxanthine). It was also prevented in mice lacking both ADO and adenosine A_2B_ receptors, suggesting that adenosine A_2B_ receptors are pronociceptive [80]. PSB-1115 treatment also decreased pain-related behaviors during the early and late phases of formalin-induced pain, suggesting peripheral and central activity of adenosine A_2B_ receptors in pain [81]. In the same study, adenosine A_3_ receptors also appeared to be pro-nociceptive. An adenosine A_3_ receptor antagonist, PSB-10, prevented the second phase of formalin-induced pain. Interestingly, it had no effect on the first phase suggesting central, but not peripheral, activity of adenosine A_3_ receptors during inflammatory pain. This pro-nociceptive effect of adenosine A_3_ receptor activation is interesting since it inhibits adenylate cyclase activity similar to that of adenosine A_1_ receptors, which are anti-nociceptive. This suggests mechanisms independent of adenylate cyclase activity that are alternatively mediated by adenosine A_1_ and A_3_ receptor activation. Contrary to this, however, central administration of an adenosine A_3_ agonist (MRS5698) was analgesic in a rat model of neuropathic pain [54]. Another A_3_ receptor agonist, IB-MECA (1-Deoxy-1-[6-[((3-Iodophenyl)methyl)amino]-9H-purin-9-yl]-N-methyl-β-D-ribofuranuronamide), also prevented the development of paclitaxel-induced neuropathic pain [54]. This may highlight a differential role of adenosine A_3_ receptors in acute (formalin) and persistent (neuropathic) pain states. These studies suggest that adenosine receptors associated with the G_s_ alpha subunit (A_2A_ and A_2B_) are generally pro-nociceptive, while those associated with the G_i_ alpha subunit (A_1_ and A_3_) are generally anti-nociceptive. This is likely due to their effects on intracellular adenylate cyclase activity and cAMP levels that have been implicated in neuropathic and inflammatory pain [82].

## 5. The Role of Adenosine in Headache Pathophysiology

Numerous key physiological changes that have been observed in chronic headache patients and animal models of trigeminal allodynia can be modulated by adenosine. This suggests that adenosine signaling may contribute to or sustain these changes. 

Mitochondrial dysfunction has been hypothesized to play a critical role in migraine. Histological studies have revealed in migraine patients ragged-red fibers and cytochrome-c-oxidase-negative fibers in skeletal muscles [83,84]. Decreased electron transport chain complex activity and metabolic abnormalities are seen in migraine patients [85,86]. We recently identified a decrease in mitochondrial spare respiratory capacity of brain sections from the TNC of the IS and STA rat models [27,87]. Mitochondrial dysfunction can affect the production of extracellular adenosine. The formation of extracellular adenosine is common in tissue hypoxia and is thought to be due to decreased mitochondrial activity such as that seen in the TNC of our models [88]. Artificially inducing mitochondrial dysfunction in isolated rat hepatocytes by treatment with the ATP synthase inhibitor, oligomycin, decreases intracellular ATP levels while increasing AMP levels [89]. Similar to adenosine production from the residual AMP molecule derived from acetate utilization in astrocytes by 5'-nucleotidases (5′NT), this increase in AMP within the cell could be a source for adenosine production when mitochondrial dysfunction is present. 5′NT activity can be enhanced during hypoxia or when ADP levels are high, increasing the production of adenosine [90,91]. ADP also increases the affinity of 5′NT for AMP, potentially facilitating the production of adenosine [92]. Mitochondrial dysfunction and adenosine signaling could affect pain via activation of AMPK, a protein kinase heavily associated with pain plasticity [93]. Extracellular adenosine, increases in the AMP:ATP ratio during hypoxia, and inhibition of 5′NT all enhance AMPK activity [94,95,96,97]. It is possible that mitochondrial dysfunction within trigeminal pain processing brain regions exacerbates adenosinergic mechanisms and AMPK activity to produce headache.

The inflammatory mechanisms thought to be involved in migraine can also be influenced by adenosine signaling. We recently found that the key component to dural-stimulated inflammatory-induced trigeminal sensitivity in rats is prostaglandin E2 (PGE2) [28]. The activation of PGE2 receptors increases intracellular cAMP levels, which is the common signaling pathway for adenosine A_2A_ and A_2B_ receptor activation. In fact, adenosine and PGE2 receptors have been shown to have synergistic effects on the suppression of the immune response of T cells [98]. PGE2 and adenosine both increase sodium channel sensitivity in neonatal rat dorsal root ganglion neurons via cAMP mediated mechanisms [99,100]. It was recently found that PGE2 may initiate its painful effects directly by increasing extracellular adenosine concentrations through PKC-mediated mechanisms [101].

The roles of glia have become more appreciated in the migraine field with their activation states potentially affecting headache [102]. Activated microglia and astrocytes could affect pain states by releasing neuroexcitatory or neuroinflammatory mediators associated with headache or even by modulating glutamatergic signaling within the brainstem [103,104,105,106]. Adenosine signaling can contribute to astrogliosis via adenosine A_2_ receptor activation but can also decrease astrogliosis via adenosine A_1_ receptor signaling [107,108]. Adenosine has profound effects on astrocytic regulation of extracellular glutamate levels. Activation of adenosine A_2A_ receptors decrease the activity of the Na/K ATPase which powers the ionic gradients required for proper glutamate transport into astrocytes through GLT1, which is responsible for deactivation and regulation of extracellular glutamate in the synaptic cleft [49,109]. This modulation is important because extracellular glutamate levels are highly associated with pain. We have found an enhanced increase in extracellular glutamate within the TNC following treatment with GTN in IS rats that can be prevented by the use of headache treatments such as noninvasive vagus nerve stimulation [26,28,29]. Similar to the TNC, modulation of glutamate regulation in the dorsal horn is associated with other forms of chronic pain [110]. Adenosine may contribute to the dysregulation of extracellular glutamate within the TNC, contributing to the development of headache.

Activation of microglia and astrocytes also modulate blood-brain barrier (BBB) permeability which has been associated with migraine patients [111,112,113,114]. Breakdown of the BBB has been hypothesized to play a key role in migraine. Serum levels of S100B, a marker for astrocyte activation and a candidate marker for changes in BBB permeability, are increased during and following migraine attacks [115]. MMP-9 and MMP-2, matrix metalloproteinases that digest endothelial cell basal lamina and cause BBB breakdown, are elevated in the serum of migraine patients [116,117,118,119]. White matter lesions in MRI images of headache patient brains have also been proposed as focal disruptions of the BBB [118,120,121]. Adenosine signaling can modulate the blood-brain barrier via changes in endothelial cells, as measured by the entrance of intravenously administered macromolecules into the brain [122]. A_1_ or A_2A_ knock-out mice feature a decrease in BBB permeability [122]. A non-selective adenosine receptor agonist decreases transendothelial electrical resistance, increases actinomyosin stress fiber formation, and alters tight junction molecules [122]. The Federal Drug Administration-approved adenosine A_2A_ agonist, Lexiscan, also increased permeability in an in vitro primary human BBB system [123]. This was further confirmed where a series of novel adenosine A_2A_ nanoagonists increased BBB permeability as measured by increased delivery of multiple drugs to the brain [124]. It is possible that repeated aberrant adenosine signaling during migraine attacks could cause long-lasting changes to BBB permeability that may contribute to headache persistence by allowing the entry of normally excluded factors into the brainstem that enhance pain hypersensitivity [125].

Furthermore, an adenosine A_2A_ receptor gene haplotype was found to be associated with migraine with aura [126]. The authors of this study ascribe this to adenosine’s regulation of the calcitonin gene-related peptide (CGRP), a neuropeptide that plays an integral role in migraine. An adenosine A_1_ receptor agonist decreased the release of CGRP and trigeminal activity in cats while an A_2A_ receptor agonist facilitated CGRP’s effect on synaptic transmission [127,128]. Interestingly, electrical stimulation of the trigeminal ganglion increased mRNA and protein levels of both CGRP and adenosine A_2A_ receptors while decreasing mRNA and protein levels of adenosine A_1_ receptors [129]. Cortical spreading depression, thought to be the cause of the aura in migraine, also induces adenosine accumulation in mice [130].

Phonophobia and photophobia are also key characteristics of migraine. Adenosine plays a significant role in both auditory and visual sensory system processing. Adenosine A_1_ receptor activation inhibits light-induced responses of intrinsically photosensitive retinal ganglion cells (ipRGCs), the non-image forming cell type that plays a critical role in exacerbating headache pain with light [131,132]. This suggests that A_1_ activation may even decrease the effects of photophobia. Furthermore, adenosine signaling plays a critical role in cochlear blood flow and the auditory system and has been investigated for its therapeutic ability to treat hearing loss [133,134].

## 6. The Role of Adenosine in Common Forms of Headache

Adenosine may play a critical role in multiple forms of headache. This observation is primarily derived from the effect of caffeine, a non-specific adenosine receptor antagonist, to relieve headache pain. In fact, a core component of many abortive headache treatments (Excedrin, Vivarin, Midol, Anacin, Migergot, and Fiorinal) is caffeine. The reasoning to include caffeine was based on the previous hypothesis that migraine was a vascular disorder and the pulsating pain associated with it was due to vasodilation of intra- or extracranial arteries [135]. Adenosine, a known vasodilator, was targeted with caffeine to decrease vasodilation. Although caffeine was found to be efficacious in headache, it is now thought that migraine headache is a neurological disorder, so caffeine’s effect as a therapeutic likely does not involve modulation of vasodilation.

Although caffeine may induce its effects on pain through other targets, clinical evidence exists for adenosine’s role in headache. Papaverine and Sildenafil, adenosine reuptake inhibitors used clinically for heart attacks, and Dipyridamole, an adenosine deaminase inhibitor used to prevent thrombosis, act by increasing extracellular adenosine levels in the brain. Interestingly, each of these drugs can also cause headache in patients [136,137]. Patients undergoing spinal anesthesia treatment to prevent pain often experience a post-operative headache. Caffeine by itself is often prescribed for treatment of this post-dural puncture headache [138]. Additionally, migraine patients without aura experience a surge in serum-adenosine levels during migraine attacks. This surge in adenosine also increases platelet uptake of serotonin, potentially accounting for the low levels of serotonin observed in migraine patients [139,140]. This process was pharmacologically determined to work through adenosine A_2_ receptors [140]. The primary mediator of adenosine receptor intracellular signaling, cAMP, which is stimulated during adenosine A_2A_ and A_2B_ receptor activation, is also suggested to play a direct role in migraine. Cilostazol, an inhibitor of phosphodiesterase type 3 which selectively degrades cAMP, produced headache in both healthy adults and migraineurs [141,142]. Below, we present numerous forms of headache and the evidence supporting the involvement of adenosine in each.

### 6.1. Delayed Ethanol-Induced Headache

Delayed ethanol-induced headache (DEIH or hangover headache) is similar to migraine headache in that it often features throbbing pain with phono- and photophobia. We previously identified that acetate, an ethanol metabolite, was the key element behind ethanol-induced trigeminal sensitivity in the IS rat model of trigeminal pain that features migraine-like characteristics [25]. In fact, clinical observations identified that the inclusion of sodium acetate as a buffer during kidney dialysis in diabetic patients also causes headache [143]. Acetate has profound effects on the formation of extracellular adenosine with many neuronal consequences of ethanol thought to be induced via acetate-mediated adenosinergic mechanisms [2]. In the brain, acetate is utilized almost exclusively by astrocytes as an energy source, resulting in a residual AMP molecule that is readily converted to adenosine [2,144]. This process is thought to be the mechanism behind the acetate-induced adenosine-mediated effects of ethanol. In humans, serum-adenosine levels rise following ethanol consumption. In wild-type rats, ethanol perfusion in the basal forebrain with microdialysis increases extracellular adenosine levels [60,144,145,146]. The headache associated with acetate is potentially due to acetate’s effect on adenosine concentrations in the brain. Not surprisingly, caffeine is widely used to treat DEIH in humans, suggesting adenosine as a key aspect to the pain phase of DEIH [147]. We also found that caffeine treatment in the IS rats prevented ethanol-induced trigeminal sensitivity [25]. Caffeine’s efficacy in the treatment of DEIH suggests adenosine receptor signaling as a critical component to the pain phase of ethanol ingestion.

### 6.2. Caffeine Withdrawal Headache

Caffeine withdrawal headaches are very common and occur when individuals decrease their daily consumption of products containing caffeine, such as coffee or tea. The headache is relieved within one hour after re-exposure to caffeine [17]. Since basal adenosine signaling is essential for proper neuronal function, chronic exposure to caffeine likely has significant compensatory effects on adenosine signaling to sustain basal adenosine function [148]. During chronic adenosine receptor antagonism with daily caffeine use, this essential basal adenosine signal could be sustained through up-regulation of adenosine receptors, increasing extracellular adenosine content, or modulation of downstream mediators of adenosine receptor signaling. Compensatory mechanisms such as these are likely at play due to the clinical observation that caffeine tolerance quickly develops in humans [149]. In fact, chronic exposure to caffeine in zebrafish increased expression of adenosine A_1_ and A_2A_ receptors, but not A_2B_ and A_3_ receptor subtypes [150]. Chronic exposure to caffeine in rats increased A_1_ receptor expression in the hippocampus [151]. Chronic exposure to caffeine in mice induces a dose-dependent increase in A_2A_ receptor expression in the striatum and shifted low-affinity A_1_ receptors to a high affinity state [152]. Chronic caffeine intake also increases plasma adenosine levels [153]. Each of these seemingly compensatory changes caused by chronic caffeine exposure could increase the adenosine signal in the absence of caffeine during the withdrawal period, contributing to caffeine withdrawal headache that is relieved by re-exposure to caffeine.

### 6.3. Cluster Headaches

Cluster headaches are a rare but intensely painful headache that is instantly debilitating [154]. One common treatment method for these attacks is the inhalation of pure oxygen [155]. Decreased oxygen supply has been associated with an increase in adenosine levels [156,157]. The effect of pure oxygen to relieve this pain may be directly tied to a reduction in adenosine levels. Platelet aggregation is impaired in cluster headache patients and could be a sign that adenosine levels are higher in these individuals since adenosine has been shown to dose-dependently inhibit platelet aggregation in humans [158,159]. Therefore, cluster headache patients may experience greater basal levels of adenosine that could contribute to the pain phase of this disorder. Increasing oxygen content could ameliorate this by decreasing adenosine content to relieve pain.

### 6.4. Sleep-Wake Cycle Related Headaches

Some forms of headache such as migraine, cluster headache, and the hypnic headache are associated with the sleep-wake cycle [17,160,161]. Adenosine plays a critical role in circadian rhythms [162]. Extracellular adenosine accumulates during sleep deprivation to promote sleep and decreases during sleep [162,163]. Caffeine is used as a stimulant by acting as an adenosine receptor antagonist to decrease adenosine’s sleep-promoting activities [162]. These forms of headache may occur due to the daily modulation of adenosine receptor expression within the brain or adenosine metabolism enzymes [164,165]. Upon waking when adenosine levels begin to rise again, there may be a partial imbalance in the adenosine receptor signal output or changes in receptor subtype-specific output, contributing to headache via adenosinergic mechanisms. Interestingly, there is even a high prevalence of sleep disorders that afflict cluster headache patients [166]. These patients may have aberrantly high levels of adenosine due to lack of sleep. Similar to cluster headaches, caffeine is used to treat hypnic headaches and has been found to be the most effective treatment option for these patients [167].

### 6.5. Post-Traumatic Headache

Post traumatic headache occurs in nearly all patients who experience mild or severe traumatic brain injury (TBI) [168]. This headache can develop further into chronic post traumatic headache. Clinical studies have found that interstitial brain adenosine levels increase during oxygen desaturation events following TBI and are thought to contribute to secondary events following TBI [169]. In the context of TBI, adenosine is thought to have a neuroprotective effect following injury and thus, use of adenosine receptor agonists are currently being investigated for TBI treatment [6]. Although adenosine may serve a role in these neuroprotective processes, they may contribute to the headache or chronic headache that develops following injury.

### 6.6. Menstrual Migraine

Menstrual migraine occurs exclusively during the first two days of menstruation in woman [17]. During this time, woman experience a sudden decrease in estrogen and progesterone levels [170]. Estradiol, an estrogen receptor-alpha ligand, was found to increase adenosine A_1_ receptor expression in a breast cancer cell line, suggesting that estrogen receptor activation can modulate adenosine receptor expression and activity [5]. Ovariectomized rats that have systemically depleted estrogen levels, also have a four-fold decrease in adenosine A_2A_ receptors [171]. In fact, birth controls that contain estrogen are also associated with headache [172]. Progesterone and estradiol were shown to increase adenosine’s depressant actions in rat cerebral cortical neurons, suggesting that these hormones could increase the adenosine signal [173]. Rats treated with progesterone were found to have lower adenosine deaminase levels, the enzyme responsible for breaking down adenosine, suggesting increased adenosine concentrations in response to progesterone treatment [174]. Although these data would suggest that estrogen or progesterone withdrawal at the start of menses would decrease adenosine levels, they demonstrate the critical cross-talk between sex hormones and the adenosinergic system which may contribute to menstrual migraine.

## 7. Adenosine and Common Headache Triggers

Headache triggers increase the probability a headache will develop in a migraine patient within 24 hours. Triggers can be dietary (alcohol, aspartame, cheese), environmental (perfumes, the *Umbellularia californica* tree, pollution), physical (extraneous activity, inactivity, exposure to intense light), or even psychological (anxiety, stress). Adenosine is involved in a number of these common headache triggers and may act as a unifying mediator for the induction of headache by them.

Glyceryl trinitrate (GTN) is a common chemical headache trigger that induces an extended headache period in chronic migraine patients, but only a short-lived headache in normal individuals [175,176]. In the IS rat model, GTN induces an extended period of trigeminal sensitivity and increased extracellular glutamate in the TNC [26,28]. GTN can potentiate the effects of adenosine on platelet aggregation, and is thought to occur through modulation of adenosine’s intracellular downstream effector molecule, cAMP [177]. Through this mechanism, GTN could potentially enhance the adenosine signal even when adenosine levels or adenosine receptor expression are not modified. GTN also has multiple effects on mitochondrial function, an organelle that has been suggested to play a role in the development of migraine [27,178,179]. GTN treatment was found to decrease the activity of complex I in the electron transport chain within isolated rat aortas, decreasing oxygen consumption [180]. During periods of decreased mitochondrial function, extracellular levels of adenosine are known to increase [88,181,182,183]. It could be through this mechanism that GTN induces trigeminal pain.

The headache tree, or *Umbellularia californica,* is known to induce severe headache attacks following inhalation of its vapors [184]. Studies in isolated rat trigeminal ganglion neurons revealed that the monoterpene ketone, umbellulone, is the chemical substance responsible for this tree’s effect on headache and that it occurs by selectively activating transient receptor potential ankyrin 1 (TRPA1) channels in the trigeminal system to induce aberrant activity that is interpreted as headache [184]. Other environmental stimulants such as cigarette smoke, chlorine, formaldehyde, and pollution are also thought to induce headache via their activation of TRPA1 or other TRP channel subtypes such as transient receptor potential vanilloid 1 (TRPV1) or transient receptor potential menthol 8 (TRPM8) [185]. Similar to adenosine A_2A_ and A_2B_ receptors, these channels increase calcium conductance via increases in cAMP intracellular concentrations. Adenosine A_1_ and A_3_ receptors reduce cAMP levels, decreasing calcium conductance [1]. Furthermore, cAMP can actively inhibit TRP channel activity [186]. In fact, caffeine inhibits human TRPA1 channels through unknown mechanisms [187]. There may be a converging modulation of intracellular cAMP levels by both TRPA1 and adenosine receptors during pain processing. Furthermore, the activation of TRPA1 channels on human odontoblasts, cells of neural crest origin, stimulates the release of ATP which can then be quickly metabolized to adenosine [188]. Similarly, TRPV4 activation can cause ATP release in primary urothelial cells [189]. ATP has also been shown to increase capsaicin-evoked currents in TRPV1 expressing HEK293 cells, suggesting that ATP or adenosine following extracellular ATP metabolism can interact with and modulate TRPV1 activity [190]. This mechanism of ATP release and metabolism to adenosine following TRP channel activation likely also occurs in the brain and may serve as a conduit between TRP channels and headache in various environmental triggers.

Anxiety and stress is a very common headache trigger. Interestingly, adenosine plays a vital role in anxiety [3,8]. Clinical and rodent data support the concept that adenosine can be both anxiogenic and anxiolytic, with A_2A_ receptors playing a critical role [8]. In support of adenosine being anxiogenic, an adenosine A_2A_ receptor antagonist reduced anxiety and stress in rodents that was caused by maternal separation or chronic unpredictable stress [191,192]. Although a genetic polymorphism of the A_2A_ receptor gene is associated with individuals diagnosed with panic disorders, the effects of these polymorphisms on receptor function have not been investigated [193,194]. In support of adenosine being anxiolytic, A_1_ or A_2A_ knock-out mice feature anxiety-like behaviors [77,195]. Furthermore, inducing stress in rats enhanced the expression of adenosine A_2A_ receptors while the overexpression of adenosine A_2A_ receptors decreased anxiety in an open field test [196]. Although not fully understood, the critical role of adenosine A_2A_ receptors in both anxiety and headache is interesting because it provides a potential common target that may play a role in both.

## 8. Conclusions

The role of adenosine in different forms of headache, headache triggers, and basic headache physiology suggests it as a core component to headache pain. Although adenosine signaling can produce differential effects on pain, depending on the receptor subtype activated, adenosine A_2A_ receptors are likely the critical subtype involved in headache pain. Adenosine signaling may initiate headache pain by modulation of intracellular cAMP production or AMPK activity that can change neuronal conductance within critical trigeminal pain processing brain regions. It may also directly contribute to pain by modulating extracellular glutamate within the synaptic cleft of tripartite synapses in the TNC or through modulation of the BBB and enhancement of gliosis. Migraineurs may be more susceptible to these adenosinergic impacts on headache due to the potential presence of mitochondrial dysfunction which could facilitate the production of excess levels of adenosine. If adenosine plays a role in headache triggers and various types of headache, it is interesting to note that systemic changes in adenosine signaling produce headache as opposed to pain across the entire body. This suggests something unique about headache pathophysiology that makes the trigeminal system more susceptible to the effects of adenosine on pain.
